# HSPA6 augments garlic extract-induced inhibition of proliferation, migration, and invasion of bladder cancer EJ cells; Implication for cell cycle dysregulation, signaling pathway alteration, and transcription factor-associated MMP-9 regulation

**DOI:** 10.1371/journal.pone.0171860

**Published:** 2017-02-10

**Authors:** Seung-Shick Shin, Jun-Hui Song, Byungdoo Hwang, Dae-Hwa Noh, Sung Lyea Park, Won Tae Kim, Sung-Soo Park, Wun-Jae Kim, Sung-Kwon Moon

**Affiliations:** 1 Department of Food Science and Nutrition, Jeju National University, Jeju, South Korea; 2 Department of Food and Nutrition, Chung-Ang University, Anseong, South Korea; 3 Department of Urology, Chungbuk National University, Cheongju, Chungbuk, South Korea; University of South Alabama Mitchell Cancer Institute, UNITED STATES

## Abstract

Although recent studies have demonstrated the anti-tumor effects of garlic extract (GE), the exact molecular mechanism is still unclear. In this study, we investigated the molecular mechanism associated with the inhibitory action of GE against bladder cancer EJ cell responses. Treatment with GE significantly inhibited proliferation of EJ cells dose-dependently through G_2_/M-phase cell cycle arrest. This G_2_/M-phase cell cycle arrest by GE was due to the activation of ATM and CHK2, which appears to inhibit phosphorylation of Cdc25C (Ser216) and Cdc2 (Thr14/Tyr15), this in turn was accompanied by down-regulation of cyclin B1 and up-regulation of p21WAF1. Furthermore, GE treatment was also found to induce phosphorylation of MAPK (ERK1/2, p38MAPK, and JNK) and AKT. In addition, GE impeded the migration and invasion of EJ cells via inhibition of MMP-9 expression followed by decreased binding activities of AP-1, Sp-1, and NF-κB motifs. Based on microarray datasets, we selected Heat shock protein A6 (HSPA6) as the most up-regulated gene responsible for the inhibitory effects of GE. Interestingly, overexpression of HSPA6 gene resulted in an augmentation effect with GE inhibiting proliferation, migration, and invasion of EJ cells. The augmentation effect of HSPA6 was verified by enhancing the induction of G_2_/M-phase-mediated ATM-CHK2-Cdc25C-p21WAF1-Cdc2 cascade, phosphorylation of MAPK and AKT signaling, and suppression of transcription factor-associated MMP-9 regulation in response to GE in EJ cells. Overall, our novel results indicate that HSPA6 reinforces the GE-mediated inhibitory effects of proliferation, migration, and invasion of EJ cells and may provide a new approach for therapeutic treatment of malignancies.

## Introduction

Bladder cancer is the most common of all human genitourinary tumors. The worldwide incidence of bladder cancer has been sharply increasing over the past 10 years [1–3]. The most lethal type of bladder malignancy is transitional cell carcinomas (TCC), such as that found in muscle invasive bladder cancer (MIBC) [3].

The G_2_/M checkpoint is controlled by regulatory proteins, including cyclin-dependent kinase 1 (CDK1, also known as Cdc2) and cyclin B1 [4]. Accumulation of cyclin B1 increases the activity of CDK1, whose activity is negatively regulated by phosphorylation of its T14/Y15 residues [4]. This inhibitory phosphorylation at T14/Y15 is removed by Cdc25C phosphatases [4]. Damages in DNA trigger the activation of the ATM pathway. Activated ATM then stimulates the activity of CHK1 and CHK2 by phosphorylation [5]. CHK1 and CHK2 subsequently phosphorylate Cdc25C which results in their chromosomal degradation [4, 5]. In addition, cumulated studies have suggested that mitogen-activated protein kinase (MAPK) and AKT signaling cascades are frequent main events involved in multiple biologic processes, such as cell proliferation, differentiation, migration, invasion, and inflammation [6]. However, recent studies have also shown that the phosphorylation of MAPK and AKT is implicated in the growth inhibition of tumor cells and leads to the induction of cell death [7, 8].

The matrix metalloproteinases (MMPs), such as MMP-2 (gelatinase A, 72 kDa gelatinase) and MMP-9 (gelatinase B, 92 kDa gelatinase), are a family of zinc-dependent endopeptidases that have been associated with the ability of tumor cells to degrade extracellular matrix (ECM) components during tumor cell invasion [9, 10]. In particular, MMP-9 is expressed in abundance in the tissue, serum, and urine of patients with TCC and correlates with muscle invasive disease [9–11]. The transcription factors, including AP-1, SP-1, and NF-κB, control MMP-9 expression by binding to the corresponding binding sites in the MMP-9 promoter region [12, 13]. Therefore, repression of expression and secretion of MMP-9 may be an effective strategy in preventing cell migration and invasion.

Heat shock proteins (HSPs), molecular chaperones guiding proper folding of other proteins, are inducible factors upon diverse stress conditions, including heat, heavy metals, organics, oxidative radicals, and chemopreventive agents [14, 15]. HSPs are classified into 6 families based on molecular size: HSP100, HSP90, HSP70, HSP60, HSP40, and small HSPs [16]. HSPs have been implicated in the biological functions of cell proliferation, cell death, apoptosis, immune system, and oncogenesis [14–16]. Suda and colleagues have recently demonstrated that DATS treatment markedly induced HSP27 protein in human monocytic U937 leukemia cells [17].

Garlic (*Allium satibum* L.) is a perennial bulb plant that belongs to the onion genus, *Allium*. Through thousands of years of human history, garlic has long been consumed as an herbal remedy as well as a condiment for flavor in cooking. Fresh or crushed garlic releases organic sulfur compounds (OSCs) such as alliin, allicin, ajoene, diallyl polysulfides, vinyldithiins, and S-allylcysteine. Alliin is a major sulfoxide responsible for the typical pungent smell of garlic [18]. When garlic is chopped or crushed, alliin is converted into a thiosulfinate, allicin, by alliinase [18]. Allicin is highly unstable and is quickly converted into other OSCs such as diallyl sulfide (DAS), diallyl disulfide (DADS), diallyl trisulfide (DATS), and other allyl polysulfides [19, 20]. These sulfides have recently been highlighted for their anti-carcinogenic properties against various cancers and have been investigated for applications as cancer prevention reagents [21–25]. However, because of the highly unstable nature of sulfoxide, it is now believed that the most critical element of garlic's anti-carcinogenic biological properties is alliin. Although enormous efforts have been made in the study of GE in human chemoprevention, there is no report on the exact molecular mechanism of garlic extract (GE) containing alliin in the proliferation, migration, and invasion of cancer cells.

In this study, we investigated the potential suppressive effects of GE in relation to cell cycle, signaling pathways, and transcription factor-mediated MMP-9 regulation in bladder cancer EJ cells. In addition, we analyzed the differentially regulated gene expression patterns following GE treatment in EJ cells using microarray technology. Finally, we identified HSPA6 as a novel main factor that regulates GE-induced anti-cancer mechanism in EJ cells.

## Materials and methods

### Materials

Garlic extract was obtained from Egarak (Busan, Korea). Polyclonal antibodies against p-Cdc2 p34 (sc-12340-R), Cdc2 p34 (sc-54), CHK2 (sc-9064), Cdc25c (sc-327), p-Cdc25c (sc12354), p21WAF1 (sc-756), p53 (sc-126), Cyclin A (sc-751), Cyclin B1 (sc-245), p-ATM (sc-47739), ATM (sc-23921), WEE1 (sc-325), HSPA6 (sc-374589) and GAPDH (sc-20357) were obtained from Santa Cruz Biotechnology Inc. (Santa Cruz, CA, USA). Polyclonal antibodies against CHK1 (2360), p-CHK1 (2341), p-CHK2 (2661), ERK (9102), p-ERK (9101), JNK (9258), p-JNK (9251), p38 MAP kinase (9212), p-p38 MAP kinase (9211), AKT (9272), and p-AKT (9271) were obtained from Cell Signaling Technology Inc. (Danvers, MA, USA). Goat anti-rabbit IgG-horseradish peroxidase (HRP) (sc-2004), goat anti-mouse IgG-HRP (sc-2005), and donkey anti-goat IgG-HRP (sc-2020) were purchased from Santa Cruz Biotechnology Inc. Western Lightning Plus-ECL was obtained from PerkinElmer, Inc. (PerkinElmer, MA, USA). U0126, SB203580, SP600125, and wortmannin, were obtained from Calbiochem (San Diego, CA). A Nuclear Extract kit and EMSA Gel Shift kit were obtained from Panomics (Fremont, CA, USA). HSPA6 cDNA was obtained from the Korea human gene bank.

### Cell cultures

The human bladder carcinoma EJ cell line was kindly provided by Dr. Wun-Jae Kim (Department of Urology, Chungbuk National University, Chungbuk, South Korea). EJ cells were grown and maintained in 1x Dulbecco's modified Eagle's medium (DMEM) (4.5 g glucose/liter) supplemented with 10% fetal calf serum, L-glutamine and antibiotics (Biological Industries, Beit Haemek, Israel) at 37°C in a 5% CO_2_ humidified incubator. Normal human urothelial cells (HUCs) were purchased from ScienCell Research Laboratories (Carlsbad, CA, USA) and maintained in the medium specific for HUCs with supplements according to the manufacturer’s protocol.

### Cell counting

Upon reaching approximately 50% confluence, cells were treated with garlic extract (0–800 μg/ml) for 24 h. Then, cells were trypsinized with 0.25% trypsin containing 0.2% EDTA (Corning, NY, USA). 50 μL of detached cells were taken and gently mixed with 50 μL of 0.4% trypan blue (Sigma-Aldrich, MO, USA). 20 μL of cells were loaded onto each chamber of hemocytometer and counted in triplicate.

### MTT cell proliferation assay

Cellular proliferation was evaluated by 3-(4,5-dimethylthiazol-2-yl)-2,5-diphenyltetrazolium bromide (MTT). Briefly, 2x10^3^ cells were plated in 96 well plates under the treatment as indicated. Cellular density was measured as absorbance at 490 nm. Experiments were performed in quadruplicates and a representative graph of three independent experiments was presented.

### Cell-cycle analysis (FACS)

2 x 10^6^ cells were plated in 100-mm culture plates and treated with garlic extract (0–800 μg/ml). After 24 h, cells were harvested and washed twice with ice-cold PBS. Cell pellets were fixed with 70% ice-cold ethanol and incubated for 20 min at 4°C. Fixed cells were then washed twice with ice-cold PBS and resuspended in 500 μL PBS. Cells were incubated with RNase (1 mg/ml) followed by propidium iodide (50 mg/ml). Phase distribution of the cell cycle was measured by FACStar flow cytometer (BD Biosciences, San Jose, CA, USA) equipped with BD Cell Fit software.

### Immunoblots

Protein lysates were prepared as described previously [13]. Protein lysate was electrophoresed on a 10% polyacrylamide gel under denaturing conditions followed by transfer to nitrocellulose membranes (Hybond, GE Healthcare Bio-Sciences, Marlborough, MA, USA). The blots were incubated for blocking with 5% (w/v) non-fat dry milk in TBS (10mM Tris-HCl (pH 8.0), 150 mM NaCl). Membranes were incubated with primary antibodies at 4°C overnight. Then secondary antibodies were incubated for 90 min. Chemiluminescence reagent kit (GE Healthcare Bio-Sciences) was used for detection. The experiments were repeated at least 3 times.

### Wound-healing migration assay

Exponentially grown cells (3x10^5^ /well) were plated in 6-well plates. Cells were pre-treated with mitomycin C (5 μg/ml, Sigma #M4287) to inhibit cell proliferation for 2 h. Then, cell surface area was scratched with a 2-mm-wide tip. After washing with 1x PBS three times, the plate was incubated with culture media in the presence or absence of garlic extract (0–800 μg/ml) for 24 h. The recovery capacity of the cells migrating into the area was measured and compared to that of the control. Cellular images were photographed using an inverted microscope with 40x magnification.

### Boyden chamber invasion assay

Invasiveness was assessed by an invasion assay kit (Cell Biolabs, San Diego, CA, USA), used in accordance with manufacturer's instructions. Briefly, 2.5x10^4^ cells were resuspended in serum-free culture medium and incubated with mitocycin C (5 μg/ml) for 2 h before being seeded in the upper chamber. Medium containing 10% FBS was added to the lower chamber as a chemo-attractant. After 24 h, cells on the lower chamber were stained and photographed.

### Gelatin zymography

EJ cells were grown in 6-well plates until they reached 90% confluence. Following this, cells were treated with garlic extract (0–800 μg/ml) for 24h. The culture medium was collected and separated in a polyacrylamide gel containing 1 mg/mL gelatin. The gel was washed with 2.5% Triton X-100 at room temperature for 2 h followed by incubation in a buffer containing 150 mM NaCl, 10 mM CaCl_2_, and 50 mM Tris–HCl, pH 7.5 at 37°C overnight. The gel was stained with Coomassie blue (0.2%) and photographed on a light box. Gelatin degrading activity of MMPs was detected as white zones.

### Transfection of cells

EJ Cells were transfected with corresponding cDNA using lipofectamine 2000 transfection reagent (Thermo Fisher Scientific, Waltham, MA, USA) in accordance with manufacturer's protocol. After incubation for the indicated time, cells were subjected to immunoblot, FACS, wound-healing migration, Boyden chamber invasion, zymographic, and EMSA assays.

### Immunofluorescence

HSPA6 gene-transfected EJ cells were fixed in 100% methanol for 20 min and incubated in 3% BSA blocking solution for 1 h at room temperature. Cells were then incubated in anti-HSPA6 (santa cruz biotechnology; 1:300) antibody for overnight, followed by incubation with secondary antibody labeled with Alexa Fluor 488 (Molecular probes, 1:400) for 2 h. Coverslips were mounted on glass slides using mounting solution containing DAPI (Immunobioscience) for 10 min. All samples were observed under a Nikon exlipse Ti fluorescence microscope (200X).

### Nuclear extract and electrophoretic mobility shift assay (EMSA)

After harvesting cells by centrifugation, EJ cells were washed and resuspended in a buffer containing 10 mM HEPES (pH 7.9), 10 mM KCl, 1 mM DTT, 0.5 mM PMSF, 0.1 mM EDTA, and 0.1 mM EGTA. After incubating on ice for 15 min, cells were vigorously mixed with 0.5% Nonidet NP-40. Following this, the nuclear pellet was collected by centrifugation and extracted with a buffer containing 20 mM HEPES (pH 7.9), 400 mM NaCl, 1 mM DTT, 1 mM PMSF, 1 mM EDTA, and 1 mM EGTA at 4°C for 15 min. Nuclear extracts (10–20 μg) were pre-incubated with 100-fold excess unlabeled oligonucleotides spanning the −79 position of MMP-9 cis element of interest at 4°C for 30 min. The sequences were as follows: AP-1, CTGACCCCTGAGTCAGCACTT; SP-1, GCCCATTCCTTCCGCCCCCAGATGAAGCAG; and NF-κB, CAGTGGAATTCCCCAGCC. Furthermore, the reaction mixture was incubated at 4°C for 20 min in a buffer (25 mM HEPES buffer (pH 7.9), 0.5 mM EDTA, 50 mM NaCl, 0.5 mM DTT, and 2.5% glycerol) with 2 μg of poly dI/dC and 5 fmol (2 × 10^4^ cpm) of a Klenow end-labeled (32^P^-ATP) 30-mer oligonucleotide spanning the DNA binding site in the MMP-9 promoter. The reaction mixture was electrophoresed using 6% polyacrylamide gel at 4°C. Following this, X-ray film was exposed to the gel overnight.

### RNA extraction for gene expression microarray analysis

Total RNA was extracted from EJ cells treated with and without garlic extract using TRIzol reagent (Thermo Fisher Scientific, Waltham, MA, USA). RNA integrity was verified by NanoDrop 1000 Spectrophotometer (NanoDrop Technologies, Wilmington, DE, USA).

### Microarray gene expression profiling

Amplified biotinylated cRNA was generated using an Illumina TotalPrep RNA Amplification Kit (Ambion Inc., Austin, TX, USA). Briefly, cDNA containing a T7 promoter sequence was synthesized with T7 Oligo(dT) Primers. Through several amplification and labeling steps, *in vitro* transcription was performed for synthesis of multiple copies of biotinylated cRNA from cDNA. Prepared cRNA was quantified by Quant-iT^™^ RiboGreenH RNA assay kit (Invitrogen-Molecular Probes, ON, Canada) using a Victor3 spectrophotometer (PerkinElmer, CT). RNA integrity of cRNA was analyzed by NanoDrop 1000 Spectrophotometer (NanoDrop Technologies, Wilmington, DE). According to manufacturer’s protocol, 1,500 ng of biotin-labeled amplified cRNAs were hybridized to an Illumina Human-6 BeadChip (48K), version 2 (Illumina, Inc., San Diego, CA). Arrays were washed and stained with Amersham fluorolink streptavidin-Cy3 (GE Healthcare Bio-Sciences, Little Chalfont, UK) in accordance with manufacturer’s instructions. The amplified signals from arrays were then scanned by an Illumina Bead Array Reader confocal scanner (BeadStation 500GXDW; Illumina, Inc., San Diego, CA).

### Statistical analysis for gene expression microarray analysis

To identify differentially expressed genes between groups treated with and without garlic extract, we carried out the hierarchical clustering analysis described previously [26]. Genes showing a *P*-value of less than 0.001 were considered to be statistically significant. To classify genes according to their biological processes and molecular functions, we utilized Ingenuity^™^ Pathways Analysis software. The significance of each function was verified by the Ingenuity Pathway Analysis Tool (version 8.8).

### Statistical analysis

Where appropriate, data was represented as a mean ± SE. Data was evaluated by factorial ANOVA and a Fisher's least significant difference test where appropriate. Statistical significance was considered at P<0.05.

## Results

### GE inhibits proliferation of bladder cancer EJ cells via induction of G_2_/M-phase cell cycle arrest

**Fig 1:**
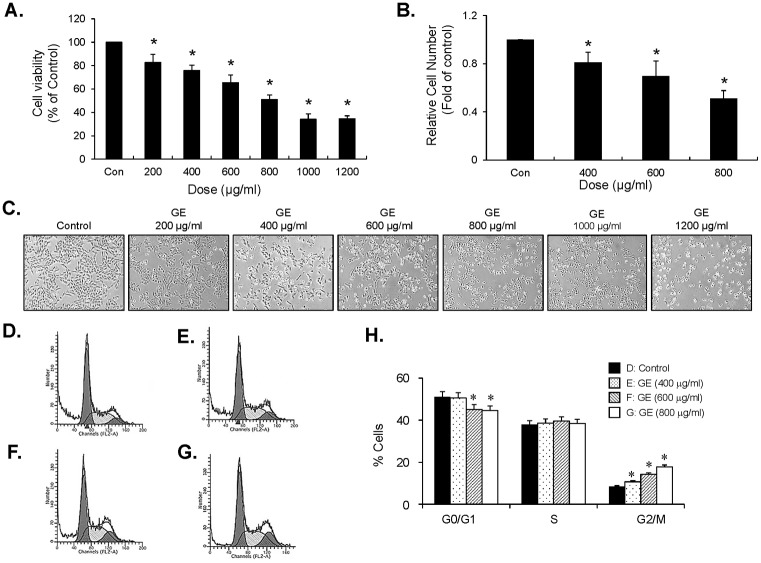
GE inhibited proliferation of EJ cells through blocking cell cycle progression at G_2_/M-phase. EJ cells were incubated in culture medium with or without GE for 24 h. Cell viability and cell proliferation was measured by both MTT (A) and viable cell counting assays (B). Results are represented as mean ± SE from three different triplicate experiments. (C) The morphology of EJ cells under different concentrations of GE. Cellular images were photographed by a phase contrast microscopy. (D-H) GE induced cell-cycle arrest at G_2_/M-phase in EJ cells. Cell cycle distribution of EJ cells treated with different concentrations of GE was determined by flow cytometry. (H) Percentage of cells in each cell-cycle phase induced by GE. Results in bar graphs are represented as mean ± SE from three different triplicate experiments. *P<0.05 compared with the control.

To investigate whether garlic extract (GE) influences the growth of bladder cancer EJ cells, cells were treated with GE (0–1200 μg/ml) for 24 h. First, the viability of cells was measured by MTT assay. As shown in Fig 1A, GE treatment showed decreased cell viability in a dose-dependent manner (Fig 1A). The dose-dependent suppression of EJ cell proliferation was verified by viable cell counting method (Fig 1B). In addition, GE treatment displayed significant morphological changes of cells which became round in shape by increasing GE concentration (Fig 1C). Based on this data, we selected an effective concentration of GE for further experimentation as lower than 800 μg/ml at which the IC_50_ concentration was observed. We, then, examined whether the cell cycle distribution was altered by GE treatment. EJ cells were treated with GE (0–800 μg/ml) for 24h, followed by flow cytometry analysis. As displayed in FACS histograms, cell populations at G_2_/M-phase increased in proportion to the concentration of GE (Fig 1D–1H). These results indicate that GE inhibited proliferation of EJ cells through inducing cell cycle arrest at the G_2_/M-phase. Furthermore, GE did not affect the viability and proliferation of normal human urothelial cells (HUC) (S1A–S1C Fig).

### GE modulates ATM-mediated CHK2/Cdc25C/Cdc2 pathway by up-regulation of p21WAF1 expression

**Fig 2:**
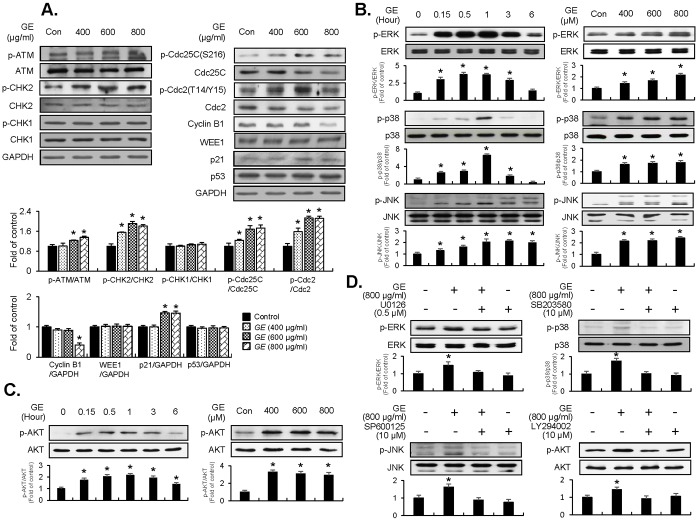
Effect of GE on G_2_/M-phase associated cell-cycle regulators and signaling pathways in EJ cells. (A) Changes in cell cycle regulators by treatment of different concentrations of GE. Immunoblots were performed using specific antibodies indicated. The bar graphs were presented as a fold ratio to the control. (B, C) Effect of GE on MAPK (ERK1/2, JNK1/2, and p38) and AKT signaling. Phosphorylation levels of each molecule were assessed by immunoblots. Bar graphs were presented as fold changes compared to the control. (D) EJ cells were pre-incubated with U0126 (0.5 μM), SB203580 (10 μM), SP600125 (10 μM), and LY 294002 (10 μM) for 40 min prior to treatment with GE (800 μg/ml). The ratio of phosphorylated to non-phosphorylated form was measured and presented as fold changes compared to the control. Results in bar graphs are represented as mean ± SE from three different triplicate experiments. *P<0.05 compared with the control.

To understand the molecular mechanism of GE on G_2_/M-checkpoint machinery, we examined effectors associated with the G_2_/M-phase cell cycle. Using protein lysates from GE-treated EJ cells, phosphorylation of ATM, CHK1/CHK2, Cdc25C, Cdc2 and expression levels of cyclin B1, wee1, p21, and p53 were analyzed by immunoblot analysis (Fig 2A). GE treatments (400, 600, and 800 μg/ml for 24 h) triggered activation of ATM and phosphorylation of CHK2 kinases, compared to the untreated control (Fig 2A, left panel). However, phosphorylation of CHK1 kinase was not observed and expression levels of total CHK1 or CHK2 proteins remained unchanged (Fig 2A, left panel). GE treatment resulted in up-regulation of inhibitory phosphorylation of Cdc25C on Ser-216. In addition, inhibitory phosphorylation of Cdc2 on T14/Y15 residues was increased and expression of cyclin B1 was reduced in GE-treated EJ cells (Fig 2A, right panel). Furthermore, p21WAF1 was up-regulated by GE, whereas the expression levels of p53 and WEE1 were unchanged (Fig 2A, right panel). These results suggest that the disruption of growth induced by GE was indeed due to the ATM-CHK2-Cdc25C-Cdc2-p21WAF1 signaling cascade in the G_2_/M checkpoint in EJ cells.

### GE treatment induces phosphorylation of MAPK (ERK1/2, JNK, and p38MAPK) and AKT

To investigate GE’s influences on the signaling pathway, we examined phosphorylation of ERK1/2, JNK, p38 MAPK, and AKT in GE-treated EJ cells using immunoblots. Treatment of GE induced phosphorylation of ERK1/2, JNK, and p38MAPK, which was sustained up to 3–6 h (Fig 2B Left panel). Treatment of cells with different concentrations of GE (400, 600, and 800 μg/ml) for 1 h resulted in a maximal increase of phosphorylation of MAPK dose-dependently (Fig 2B Right panel). Moreover, GE treatment significantly up-regulated AKT phosphorylation compared to the control (Fig 2C Right panel). Time-course induction of AKT phosphorylation showed that the signal was peaked at 1 h after GE treatment (Fig 2C Left panel). These results were verified by utilizing corresponding inhibitors: SP600125 (an inhibitor of JNK), U0126 (an inhibitor of ERK1/2), SB203580 (an inhibitor of p38MAPK), or LY294002 (an inhibitor of AKT). As shown in Fig 2D, pre-incubation of EJ cells with U0126, SP600125, SB203580, or LY294002 reversed GE-induced phosphorylation of ERK1/2, JNK, p38MAPK, and AKT, respectively (Fig 2D). These results indicate that treatment of GE up-regulates both MAPKs (ERK, JNK, and p38MAPK) and AKT. To investigate whether these effectors are sufficient in GE-induced inhibition of EJ cell proliferation, we utilized inhibitors for each kinase (S2A–S2C Fig). Interestingly, U0126, SP600125, SB203580, or LY294002 showed no effects on viability and morphology of EJ cells (S2A–S2C Fig). These results suggest that MAPKs and AKT are required but not sufficient in GE-induced inhibition of proliferation of EJ cells.

### GE inhibits migration and invasion of EJ cells through diminished MMP-9 expression by reduction of AP-1, Sp-1, and NF-κB binding activities

**Fig 3:**
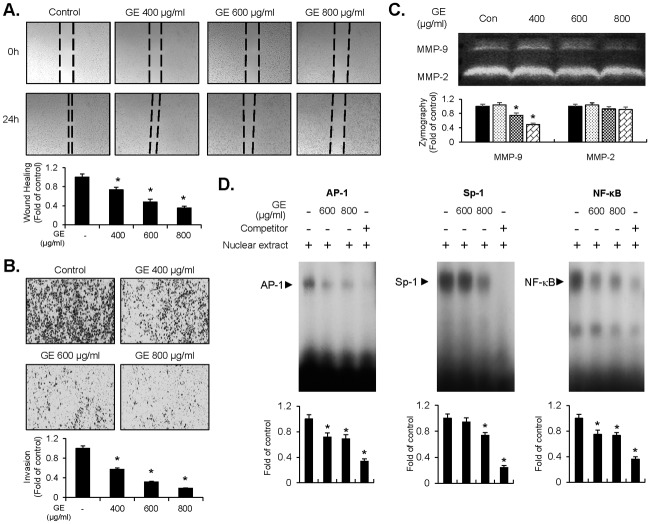
GE inhibited the migration and invasion of EJ cells through diminished MMP-9 activity by suppressing binding activity of transcription factor AP-1, Sp-1, and NF-κB. (A) Changes of migratory potential were assessed by scratch wound-healing assays. The cells were pre-treated with mitocycin C (5 μg/ml) for 2 h. The surface area of migrating cells was photographed by a phase contrast microscope. The recovery rate was measured as fold changes compared with the control. (B) Invasive capacity was measured using Matrigel^®^-coated transwell plates in GE-treated EJ cells. The cells were incubated with mitocycin C (5 μg/ml) for 2 h before the invasion assays. Cellular images were taken by a phase contrast microscope. The amount of invading cells was presented as a fold change relative to the control. (C) Gelatinase activity of MMP-2 and -9 were assessed by different concentrations of GE using zymography. Bar graph was presented as a fold change contrasted with the control. (D) Binding activity of transcription factor AP-1, Sp-1, and NF-κB, was measured by EMSA in GE-treated EJ cells. Bar graph was presented as a fold change compared with the control. Results in bar graphs are represented as mean ± SE from three different triplicate experiments. *P<0.05 compared with the control.

The next focus of research was whether GE attenuates the migratory and invasive capacity of EJ cells. Cells, grown up to 90% confluence, were scratched with a pipette tip and incubated with medium in the presence or absence of GE (400–800 μg/ml) for 24h. As exhibited in Fig 3A, recovery rate of the scratched area was decreased by GE treatment dose-dependently. In addition, alteration in the invasive potential of EJ cells was measured with a trans-well invasion assay using a gelatin-coated membrane. GE treatment resulted in a reduction of EJ cell invasion through the membrane in a dose-dependent manner, as compared to the control (Fig 3B). However, GE treatment did not affect the migration and invasion of HUC cells (S1D–S1E Fig). These results demonstrate that GE may successfully suppress the metastatic activity of bladder cancer cells. Recently, several studies have reported that the expression of MMP-9 is associated with migration, invasion, and aggressiveness of tumor cells [27, 28]. Thus, we examined whether GE inhibits the activity of MMP-2 and -9 in EJ cells. The proteinase activity of MMP-9 was reduced by GE treatment, but had no effect on MMP-2 (Fig 3C). Subsequently, we investigated the transcription factors responsible for the inhibition of MMP-9 activity. To this end, we carried out on electrophoretic mobility shift assay (EMSA) using oligonucleotides of transcription factors spanning sequences of the MMP-9 cis-element. As shown in Fig 3D, binding activities of transcription factors, AP-1, Sp-1, and NF-κB, were reduced by the treatment of GE. These findings suggest that GE inhibits the migratory and invasive ability of EJ cells through the suppression of MMP-9 activity, which is represented by hampering binding activities of AP-1, Sp-1, and NF-κB motifs.

### Microarray analysis indicates different gene expression patterns in GE-treated EJ cells

**Fig 4:**
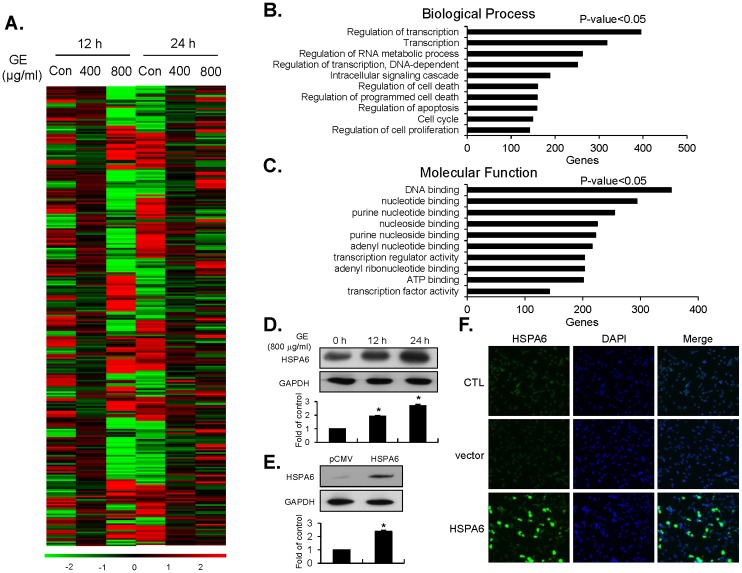
Gene expression patterns in EJ cells treated with GE. (A) Clustergram of differentially expressed genes by GE treatment was produced using microarray analysis. The red and green colors indicate high and low expression of particular genes, respectively. (B, C) Differentially expressed genes upon GE treatments were categorized by their biological processes (BP) and molecular functions (MF) through GO term enrichment analysis. (D) The expression of HSPA6 in response to GE treatment at different time points was measured by immunoblots. (E) Transfection efficiency of HSPA6 cDNA gene was evaluated by transient transfection compared to an empty vector (EV), pCMV. (F) After transfection of the HSPA6 gene, expression levels of HSPA6 were confirmed by immunofluorescence.

To identify key molecular targets, we performed microarray analysis with RNAs extracted from EJ cells treated with or without GE (400 and 800 μg/ml), by which expression strength is distinguishable over the 47,323 target sequences derived from RefSeq genes. A clustergram of the microarray data showed that genes are differentially expressed by incubation periods (12 h and 24 h) and concentrations of GE (0, 400, and 800 μg/ml) (Fig 4A). In order to classify genes in terms of their functions and significance, we utilized the DAVID database. First, we performed a Gene Ontology (GO) enrichment analysis on differentially expressed gene sets, according to classification in both biological process (BP) and molecular function (MF). BP analysis generated the 10 most significant terms as the following: regulation of transcription, transcription, regulation of RNA metabolic process, regulation of DNA-dependent transcription, intracellular signaling cascade, regulation of cell death, regulation of programmed cell death, regulation of apoptosis, cell cycle, and regulation of proliferation (Fig 4B). MF analysis showed 10 most significant terms: DNA binding, nucleotide binding, purine nucleotide binding, nucleoside binding, purine nucleoside binding, adenyl nucleotide binding, transcription regulator activity, adenyl ribonucleotide binding, ATP binding, and transcription factor activity (Fig 4C). By BP analysis, we selected the top 10 most up-regulated genes (ATF3, ID2, CRYAB, RASD1, HEY1, HSPA1B, HSPA1A, RGS2, GADD45B, and HBEGF) and the top 10 most down-regulated genes (SMAD3, MAP3K1, THBS1, CCL2, CEBPD, FAS, CITED4, TNS3, CCND2, and EFNA1) in GE-treated groups, compared to the control (Tables 1 and 2). Through MF analysis, the top 10 most up-regulated genes (HSPA6, HSPA1B, HSPA1A, GEM, ATF3, HEY3, DDIT3, MXD1, ASD1, and ID2) and down-regulated genes (SMAD3, MAP3K1, CEBPD, TAF15, TRIB2, RIPK4, ZFP36L2, D2HGDH, GBP1, and CITED4) were selected (Tables 3 and 4). Subsequently, by using a fold change filter, we identified the 11 most up-regulated genes (Table 5). Because HSPA6 was the most highly up-regulated gene as a result of GE treatment, HSPA6 was selected for further investigation. In order to verify the result of the microarray data, we examined whether the expression of HSPA6 protein was up-regulated by GE treatment in different periods (12 h and 24 h). As shown in Fig 4D, HASPA6 protein was markedly increased by GE treatment (Fig 4D). For more details on microarray data, please refer to S1 Table.

**Table 1:** The 10 most up-regulated genes found with BP analysis following GE treatment.

Symbol	Description	Gene Ontology Term
*ATF3*	Activating transcription factor 3	Regulation of transcription,
		Regulation of cell proliferation,
		Transcription,
		Regulation of RNA metabolic process,
		Regulation of transcription, DNA-dependent
*ID2*	Inhibitor of DNA binding 2, dominant negative helix-loop-helix protein	Regulation of transcription,
		Regulation of cell proliferation,
		Regulation of RNA metabolic process,
		Regulation of transcription, DNA-dependent
*CRYAB*	Crystallin, alpha B	Intracellular signaling cascade,
		Regulation of cell death,
		Regulation of programmed cell death,
		Regulation of apoptosis
*RASD1*	RAS, dexamethasone-induced 1	Regulation of transcription,
		Regulation of RNA metabolic process,
		Regulation of transcription, DNA-dependent,
		Intracellular signaling cascade
*HEY1*	Hairy/enhancer-of-split related with YRPW motif 1	Regulation of transcription,
		Transcription,
		Regulation of RNA metabolic process,
		Regulation of transcription, DNA-dependent
*HSPA1B*	Heat shock 70kDa protein 1A; heat shock 70kDa protein 1B	Regulation of cell death,
		Regulation of programmed cell death,
		Regulation of apoptosis
*HSPA1A*	Heat shock 70kDa protein 1A; heat shock 70kDa protein 1B	Regulation of cell death,
		Regulation of programmed cell death,
		Regulation of apoptosis
*RGS2*	Regulator of G-protein signaling 2	Cell cycle
*GADD45B*	Growth arrest and DNA-damage-inducible, beta	Intracellular signaling cascade
*HBEGF*	Heparin-binding EGF-like growth factor	Regulation of cell proliferation

**Table 2:** The 10 most down-regulated genes found with BP analysis following GE treatment.

Symbol	Description	Gene Ontology Term
SMAD3	SMAD family member 3	Regulation of transcription
		Regulation of cell proliferation
		Transcription
		Regulation of RNA metabolic process
		Regulation of transcription, DNA-dependent
		Regulation of cell death
		Regulation of programmed cell death
		Regulation of apoptosis
		Cell cycle
MAP3K1	Mitogen-activated protein kinase kinase kinase 1	Regulation of transcription
		Regulation of RNA metabolic process
		Regulation of transcription, DNA-dependent
		Intracellular signaling cascade
		Regulation of cell death
		Regulation of programmed cell death
		Regulation of apoptosis
THBS1	Thrombospondin 1	Regulation of cell proliferation
		Intracellular signaling cascade
		Regulation of cell death
		Regulation of programmed cell death
		Regulation of apoptosis
		Cell cycle
CCL2	Chemokine (C-C motif) ligand 2	Regulation of cell proliferation
		Intracellular signaling cascade
		Regulation of cell death
		Regulation of programmed cell death
		Regulation of apoptosis
CEBPD	CCAAT/enhancer binding protein (C/EBP), delta	Regulation of transcription
		Transcription
		Regulation of RNA metabolic process
		Regulation of transcription, DNA-dependent
FAS	Fas (TNF receptor superfamily, member 6)	Regulation of cell death
		Regulation of programmed cell death
		Regulation of apoptosis
CITED4	Cbp/p300-interacting transactivator,	Regulation of transcription
		Transcription
TNS3	Tensin 3	Regulation of cell proliferation
		Intracellular signaling cascade
CCND2	Cyclin D2	Regulation of cell proliferation
		Cell cycle
EFNA1	Ephrin-A1	Intracellular signaling cascade

**Table 3:** The 10 most up-regulated genes found with MF analysis following GE treatment.

Symbol	Description	Gene Ontology Term
HSPA6	heat shock 70kDa protein 6 (HSP70B')	Nucleotide binding
		Purine nucleotide binding
		Nucleoside binding
		Purine nucleoside binding
		Adenyl nucleotide binding
		Adenyl ribonucleotide binding
		ATP binding
HSPA1B	Heat shock 70kDa protein 1B	Nucleotide binding
		Purine nucleotide binding
		Nucleoside binding
		Purine nucleoside binding
		Adenyl nucleotide binding
		Adenyl ribonucleotide binding
		ATP binding
HSPA1A	Heat shock 70kDa protein 1A	Nucleotide binding
		Purine nucleotide binding
		Nucleoside binding
		Purine nucleoside binding
		Adenyl nucleotide binding
		Adenyl ribonucleotide binding
		ATP binding
GEM	GTP binding protein overexpressed in skeletal muscle	Nucleotide binding
		Purine nucleotide binding
		Nucleoside binding
		Purine nucleoside binding
ATF3	Activating transcription factor 3	DNA binding
		Transcription factor activity
		Transcription regulator activity
HEY1	Hairy/enhancer-of-split related with YRPW motif 1	DNA binding
		Transcription factor activity
		Transcription regulator activity
DDIT3	DNA-damage-inducible transcript 3	DNA binding
		Transcription factor activity
		Transcription regulator activity
MXD1	MAX dimerization protein 1	Transcription factor activity
		Transcription regulator activity
ASD1	RAS, dexamethasone-induced 1	Nucleotide binding
		Purine nucleotide binding
ID2	Inhibitor of DNA binding 2, dominant negative helix-loop-helix protein	Transcription regulator activity

**Table 4:** The 10 most down-regulated genes found with MF analysis following GE treatment.

Symbol	Description	Gene Ontology Term
SMAD3	SMAD family member 3	DNA binding
		Transcription factor activity
		Transcription regulator activity
MAP3K1	Mitogen-activated protein kinase kinase kinase 1	Nucleotide binding
		Purine nucleotide binding
		Nucleoside binding
		Purine nucleoside binding
		Adenyl nucleotide binding
		Adenyl ribonucleotide binding
		ATP binding
CEBPD	CCAAT/enhancer binding protein (C/EBP), delta	DNA binding
		Transcription factor activity
		Transcription regulator activity
TAF15	TAF15 RNA polymerase II, TATA box binding protein (TBP)-associated factor, 68kDa	DNA binding
		Nucleotide binding
		DNA binding
		Nucleotide binding
TRIB2	Tribbles homolog 2 (Drosophila)	Nucleotide binding
		Purine nucleotide binding
		Nucleoside binding
		Purine nucleoside binding
		Adenyl nucleotide binding
		Adenyl ribonucleotide binding
		ATP binding
RIPK4	Receptor-interacting serine-threonine kinase 4	Nucleotide binding
		Purine nucleotide binding
		Nucleoside binding
		Purine nucleoside binding
		Adenyl nucleotide binding
		Adenyl ribonucleotide binding
		ATP binding
ZFP36L2	Zinc finger protein 36, C3H type-like 2	DNA binding
		Transcription factor activity
		Transcription regulator activity
D2HGDH	D-2-hydroxyglutarate dehydrogenase	Nucleotide binding
		Purine nucleotide binding
		Nucleoside binding
		Purine nucleoside binding
		Adenyl nucleotide binding
GBP1	Guanylate binding protein 1, interferon-inducible, 67kDa	Nucleotide binding
		Purine nucleotide binding
CITED4	Cbp/p300-interacting transactivator, with Glu/Asp-rich carboxy-terminal domain, 4	Transcription regulator activity

**Table 5:** Up-regulated genes following GE treatment (800 μg/ml).

Gene name	NCBI Genebank accession no.	Fold change	
		12h	24h
*CRYAB*	NM_001885.1	27.695435	26.308425
*HSPA6*	NM_002155.3	122.90599	37.306109
*ID2*	NM_002166.4	34.328057	17.260472
*ID2*	NM_002166.4	31.948792	15.607606
*LOC100132564*	XM_001713808.1	34.515068	44.478887
*RN5S9*	NR_023371.1	82.462446	77.444412
*RN7SK*	NR_001445.1	88.432765	49.193239
*RN7SK*	NR_001445.1	34.038295	22.660076
*RNU1-3*	NR_004408.1	36.466343	14.503628
*RNU1-5*	NR_004400.1	32.151429	12.610301
*RNU1G2*	NR_004426.1	37.858887	13.874181
*SNORD3A*	NR_006880.1	7.476308	14.529322
*SNORD3D*	NR_006882.1	8.933449	16.526395

### Overexpression of HSPA6 enhances the inhibitory effect of GE on the proliferation, migration, and invasion of EJ cells

**Fig 5:**
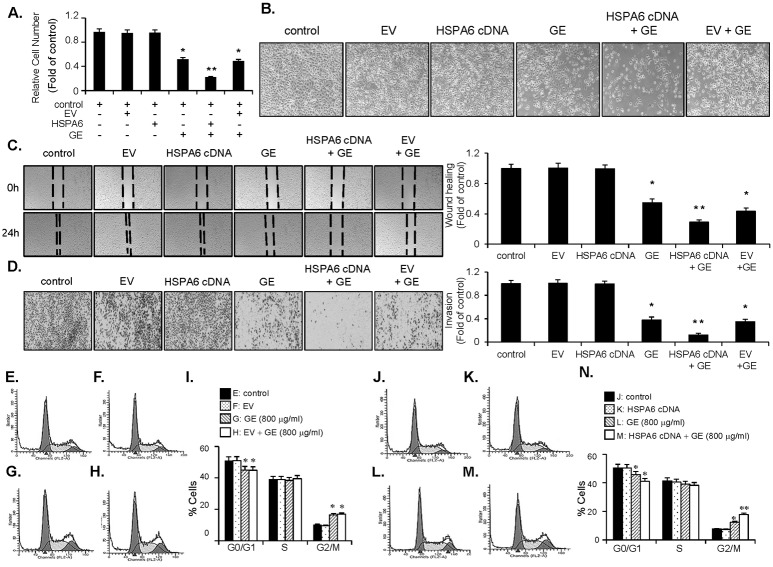
HSPA6 potentiated the suppression of proliferation, migration, and invasion and the accumulation at G_2_/M cell-cycle phase in GE-treated EJ cells. After transfection of either HSPA6 or an EV, cells were incubated with or without GE (800 μg/ml). (A) Effect of HSPA6 gene in GE-mediated inhibition of proliferation of EJ cells. Relative cell number was expressed as a fold change compared with the control. (B) Images of cellular morphology were photographed by a phase contrast microscope. (C) Effect of HSPA6 gene on GE-mediated inhibition of migration. After incubation of mitocycin C (5 μg/ml) for 2 h, wound recovery capacity was evaluated by comparing migrated area after 24 h. (D) Effect of HSPA6 on GE-mediated inhibitory activity against invasive potential of EJ cells. The cells were incubated with mitocycin C (5 μg/ml) for 2 h before the invasion assay. Cellular images were photographed by a phase contrast microscope after staining with crystal violet. (E-N) Effects of HSPA6 gene in GE-mediated G_2_/M-phase cell cycle distribution. GE-mediated G_2_/M cell-cycle phase distribution was evaluated by flow cytometric analysis. Percentage of cell populations in each cell-cycle phase was presented by bar graphs. Results in bar graphs are shown as a mean ± SE from three different triplicate experiments.*P < 0.05, compared with the control and **P < 0.05, compared with GE treatment.

The next investigation focused on whether overexpression of the gene mimics the inhibitory effects of GE on the proliferation of EJ cells. To this end, EJ cells were transfected with HSPA6 or on an empty vector (EV), followed by incubation with culture media in the presence or the absence of GE (800 μg/ml). Firstly, we confirmed the transfection efficiency of HSPA6 using an immunoblot and an immunofluorescene (Fig 4E and 4F). As shown in Fig 5A, the inhibitory effect in the proliferation of EJ cells was not observed by transfection of HSPA6 alone, compared to the control or the EV-transfectant. Although HSPA6 alone never showed growth suppression, notably, overexpression of HSPA6 showed a potentiating effect with GE on the inhibition of the proliferation of EJ cells, compared with either cells treated with GE or EV-transfected cells treated with GE (Fig 5A). Cellular images of HSPA6-transfectants supported the notion that HSPA6 indeed enhanced the inhibitory effect of GE (Fig 5B). Subsequently, we examined whether introduction of the HSPA6 gene affected the migratory and invasive potential of EJ cells. As demonstrated in Fig 5C and 5D, HSPA6 did not show suppressive effects in the migration and invasion of EJ cells. However, transfection of HSPA6 exhibited a potentiating effect with GE treatment, but neither GE alone nor the GE-treated EV transfectants showed the effect (Fig 5C and 5D). Our data indicates that the overexpression of the HSPA6 gene fortified the inhibitory effect of GE on the proliferation, migration, and invasion of EJ cells.

### HSPA6 augments the changed expression level of G_2_/M-phase cell cycle regulators and phosphorylation of signaling pathways in GE-treated EJ cells

**Fig 6:**
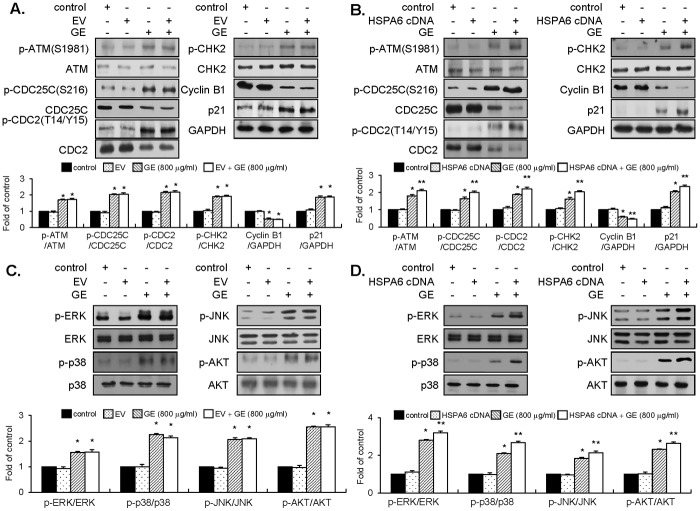
HSPA6 gene enhanced regulatory proteins participated in GE-mediated G_2_/M-phase cell cycle and early signaling pathways in EJ cells. EJ cells were transfected with an EV (A, C) or HSPA6 (B, D) followed by incubation in the culture medium with or without GE. Cell cycle regulators and signaling molecules were assessed by immunoblots using specific antibodies indicated. Expression levels of proteins were normalized by corresponding total forms or GAPDH. *P < 0.05, compared with the control and **P < 0.05, compared with GE treatment.

Because HSPA6 potentiated the inhibition of proliferation of EJ cells induced by GE, we investigated whether HSPA6 affects G_2_/M-phase cell-cycle machinery and signaling pathways in GE-treated EJ cells. GE treatment of HSPA6-transfectants revealed more accumulation in G2/M-phase cell cycle than that of GE alone (Fig 5J–5N). However, EV-transfected cells were not affected by GE treatment (Fig 5E–5I). We next performed immunoblots to examine the alteration of expression level of G_2_/M-phase-associated proteins by introducing the HSPA6 gene in GE-treated cells. Overexpression of HSPA6 in EJ cells significantly reinforced induction of the ATM-mediated CHK2/Cdc25C/Cdc2 signaling induced by GE (Fig 6B). However, EV-transfectants did not affect the GE-mediated induction of the ATM/CHK2/Cdc25C/Cdc2 signaling cascade (Fig 6A). In addition, transfection of HSPA6 followed by GE treatment showed a potentiation effect in either up-regulation of p21WAF1or down-regulation of cyclin B1 (Fig 6B) which was not seen in EV-transfectants (Fig 6A). Furthermore, phosphorylation of ERK1/2, JNK, p38, and AKT by GE was remarkably fortified in the presence of HSPA6 gene, compared with GE alone (Fig 6D). However, EJ cells transfected with an EV showed no effect on GE-mediated signaling pathways (Fig 6C). These results suggest that HSPA6 may be a key regulator that participates in the inhibition of proliferation of EJ cells by GE through dysregulation of G_2_/M-phase-associated proteins and control of signaling pathways.

### Overexpression of HSPA6 intensifies the inhibitory potential of GE on MMP-9 expression via binding activities of AP-1, Sp-1, and NF-κB in EJ cells

**Fig 7:**
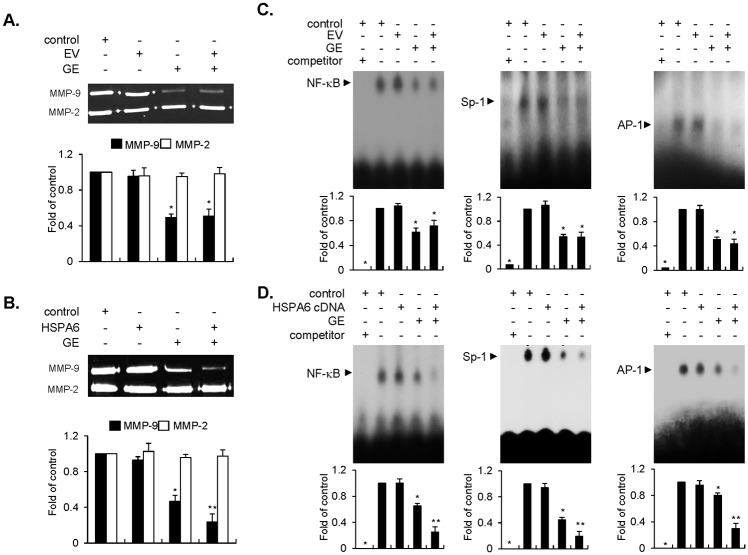
HSPA6 intensified GE-mediated inhibitory effect of MMP-9 activity via suppression of binding activity of AP-1, Sp-1, and NF-κB in EJ cells. EJ cells were transfected with an EV (A, C) or HSPA6 (B, D) followed by incubation either in the presence or absence of GE. (A, B) Proteolytic activity of MMP-9 was assessed by gelatin zymography. (C, D) EMSA was performed to detect the binding activity of AP-1, Sp-1, and NF-κB using radiolabeled oligonucleotide probes. Unlabeled AP-1, Sp-1, and NF-κB oligonucleotides were used as competitors. Relative fold changes were indicated to compare against the control versus GE treatment. In each bar graph, results are presented as the mean ± SE from three different triplicate experiments.*P < 0.05, compared with the control and **P < 0.05, compared with GE treatment.

Since HSPA6 enhanced GE-induced inhibition of migration and invasion of EJ cells, we examined whether HSPA6 affects the enzymatic activity of MMPs using gelatin zymography. Introduction of the HSPA6 gene augmented the inhibitory activity of MMP-9 enzyme induced by GE, compared to GE alone (Fig 7B). On the contrary, similar level of MMP-9 enzyme activity was observed in both GE alone and GE treatment of EV-transfectants (Fig 7A). Finally, the present results from EMSA demonstrated that diminished binding activities of transcription factors (AP-1, Sp-1, and NF-κB) were further amplified by transfection of the HSPA6 gene in GE-treated cells (Fig 7D), compared to cells transfected with EV (Fig 7C). These results indicate that HSPA6 might be a critical regulator in governing MMP-9 activity via inhibition of the transcriptional binding activity of AP-1, Sp-1, and NF-κB motifs in GE-treated EJ cells.

## Discussion

In this study, we investigated the molecular mechanisms of garlic extract (GE) in regulation of proliferation, migration, and invasion of bladder cancer EJ cells. Additionally, microarray analysis was employed to identify differentially regulated genes in response to GE in EJ cells. Furthermore, based on array datasets, we discovered a novel role of heat shock protein A6 (HSPA6) that is involved in the GE-mediated inhibition of proliferation, migration, and invasion of EJ cells associated with cell cycle dysregulation, signaling pathway alteration, and transcription factor-connected MMP-9 regulation.

It is well known that GE leads to apoptosis in diverse cancer cells, which suggests GE as a potential chemo-preventive reagent [21–25]. However, the exact molecular mechanism underlying inhibition of proliferation, migration, and invasion induced by GE is largely unknown. Therefore, to better understand the mode of molecular action of GE, we investigated how GE controls various molecular regulators in bladder cancer EJ cells. Treatment of GE showed a significant inhibition in proliferation of EJ cells, but not in normal HUC cells, via inducing G_2_/M-phase cell cycle arrest. It is well known that the complex between Cdc2 and cyclin B1 is important for entry into mitosis in most organisms [4, 29]. The activity of Cdc2/cyclin B1 kinase is negatively regulated by reversible phosphorylations at Thr14 and Tyr15 of Cdc2 [4, 29]. Dephosphorylation of Thr14 and Tyr15 of Cdc2, and hence activation of the Cdc2/cyclin B1 kinase complex is catalyzed by the Cdc25 family of dual specificity phosphatases, it is this reaction which is believed to be a rate-limiting step for entry into mitosis [4, 29]. In the present study, treatment of GE induced activation of ATM and phosphorylation of CHK2 kinases, which resulted in the inhibitory phosphorylation of Cdc25C on Ser-216 and up-regulation of p21WAF1 expression. Subsequently, an inhibitory phosphorylation of Cdc2 on Thr-14 and Tyr-15 was increased and the expression levels of cyclin B1 were decreased in GE-treated EJ cells. Consequently, our results show that GE-stimulated G_2_/M-phase cell cycle arrest was followed by ATM-mediated phosphorylation of CHK2-Cdc25C-Cdc2 pathway and induction of p21WAF1 expression, which was the basis for the inhibition of proliferation of EJ cells induced by GE.

We expanded investigation into MAPK and AKT signaling pathways due to MAPK and AKT signaling causing remarkable suppression of the proliferation in cancer cells [6–8]. Previous study had suggested that GE inhibited the proliferation of human endometrial cells via inhibiting ERK1/2 and JNK1/2 [30]. In other research, aged black garlic extract inhibited proliferation of HT-29 colon cancer cells by down-regulating AKT activity [31]. In disagreement with the previous results, we demonstrated that GE treatment induced phosphorylation of ERK1/2, JNK, p38, and AKT. However, the signaling inhibitors did not recover the reduced proliferation of EJ induced by GE, suggesting that MAPK and AKT signaling would not be sufficient to affect the proliferation of EJ cells. Future study will be remained to examine the exact mechanism of the signaling pathway in GE-mediated anti-proliferation in EJ cells.

It has been well demonstrated that metastases formation is one of the critical factors in managing cancer patients as it accounts for the majority of cancer deaths [9, 10]. Therefore, it has been proposed that targeting the migratory and invasive potential of tumor cells could be an effective tactic in treating muscle invasive bladder cancer (MIBC) [9, 10, 32]. Accordingly, we investigated whether GE suppresses the migratory and invasive activity of bladder cancer cells. In the present study, GE treatment impeded the migration and invasion of bladder cancer EJ cells. However, the migratory and invasive potential was not affected in HUC cells following treatment with GE. In addition, the results from the gelatin zymography suggest that GE-mediated inhibition of proliferation in EJ cells was due at least in part to the inhibition of MMP-9 activity but not MMP-2. In concurrence with our results, it has been demonstrated that garlic-derived diallyl sulfide (DAS) disrupts MMP-9 expression in human colon cancer cells [25]. Furthermore, the regulatory mechanism of MMP-9 in GE-treated cells was investigated because the migration and invasion of tumor cells is connected with MMP-9 expression through the control of transcription factors AP-1, Sp-1, and NF-κB [12, 13, 32]. Treatment of EJ cells with GE markedly down-regulated the binding activities of AP-1, Sp-1, and NF-κB. Our findings indicate that GE inhibited MMP-9 expression via reduction of the binding activity of transcription factor AP-1, Sp-1, and NF-κB, leads to the repression of migration and invasion of EJ cells.

Although many studies have demonstrated the anti-tumor effects of GE, the main molecular genes that affect this action still remain largely unknown. To identify molecular markers responsible for the inhibitory activity of GE, we employed microarray technology. After profiling differentially expressed genes between GE-treated and untreated EJ cells, we selected the top 10 up-regulated and the top 10 down-regulated genes in both BP and MF categories (Tables 1–4). We identified, for the first time, 40 genes associated with the inhibitory activity of GE against proliferation, migration, and invasion of EJ cells. Moreover, based on screening by fold changes, we picked up 11 candidates that are up-regulated differentially by GE treatment (Table 5). Among these genes, we eventually identified that HSPA6 contributes to the inhibitory effect of GE on proliferation, migration, and invasion of EJ cells. After transient transfection, we found that HSPA6 indeed is essential as an augment factor which potentiates the inhibitory effects of GE on the proliferation of EJ cells, which was due to the G2/M-phase cell cycle arrest induced by ATM/CHK2/Cdc25C/p21WAF1/Cdc2 cascade and induction of phosphorylation of MAPK (ERK1/2, JNK, and p38MAPK) and AKT signaling. Furthermore, we also verified that HSPA6 further intensifies the GE-induced disruption of migration and invasion of EJ cells caused by a decrease in MMP-9 activity through a suppressive effect on transcription factors (AP-1, Sp-1, and NF-κB). Collectively, our results demonstrate that HSPA6 may act as an important augmentation factor, which contributes to the decreased proliferation, migration, and invasion found in EJ cells treated with GE.

HSPA6, also known as HSP70B, belongs to the HSP70 family. Although the exact function of HSPA6 is unclear, it has been reported that expression of HSPA6 is essential for improving cell survival under extracellular stress such as high temperature or exposure to toxicity [14–16, 33, 34]. Previous studies demonstrated that HSPA6 expression exhibited stress-protective functions in neuronal cells and keratinocytes [33, 34]. However, in disagreement with previous results, our data showed that HSPA6 enhanced GE-induced inhibition of cellular physiology, including proliferation, migration, and invasion, in bladder cancer EJ cells. These results are a significant step forward in understanding the function of HSPA6 in GE’s anti-tumor effect, and it also provides new insight on the molecular mechanisms underlying the unexpected effects of HSPA6. Although our data has demonstrated the importance of the HSPA6 in GE-treated EJ cells, additional studies should be performed to clearly elucidate the relationship between other HSP family molecules and the anti-tumor effect of GE.

In conclusion, this study elucidates on the molecular mechanism of the GE-induced inhibition of proliferation, migration, and invasion of EJ cells, which is involved in cell cycle, signaling pathway, and MMP-9 regulation by transcription factors. In addition, we identified the differential gene expression patterns in GE-treated EJ cells. Furthermore, we found a novel role of HSPA6 functioning as a potentiation regulator in the GE-mediated anti-tumor effect, which suggests a capability of prognostic marker for enhancing the GE-induced anti-tumor effects. Further study is necessary to examine the combinational anti-tumor efficacy of HSPA6 gene and GE using a bladder cancer-derived xenograft animal model.

## Supporting information

S1 Table Microarray dataset on differentially expressed genes in response to GE treatment. (XLSX)

S1 Fig GE did not affect the proliferation, migration, and invasion of HUC cells. HUC cells were cultured with or without GE for 24 h. The cell viability and cell proliferation was estimated by both MTT (A) and viable cell counting assay (B). Results are expressed as mean ± SE from three different triplicate experiments. (C) The cell morphology generated from different concentrations of GE. Cellular images were captured with a phase contrast microscopy. (D, E) Wound-healing migration and invasion assay following treatment with GE in HUC cells. Results in bar graphs are expressed as mean ± SE from three different triplicate experiments.
(TIFF)

S2 Fig Inhibition of signaling pathway had no effect in the GE-induced anti-proliferation of EJ cells. EJ cells were pre-treated with U0126 (0.5 μM), SB203580 (10 μM), SP600125 (10 μM), and LY 294002 (10 μM) for 40 min following treatment with GE (800 μg/ml). MTT (A) and viable cell counting assay (B) were performed to determine the cell viability and cell proliferation. (C) The cell morphology was photographed using a phase contrast microscopy. Results in bar graphs are reported as mean ± SE from three different triplicate experiments. *P<0.05 compared with the control.
(TIFF)
