# Supplementary figures and images for: HSPA6 augments garlic extract-induced inhibition of proliferation, migration, and invasion of bladder cancer EJ cells; Implication for cell cycle dysregulation, signaling pathway alteration, and transcription factor-associated MMP-9 regulation
Source: PLoS One. 2017 Feb 10;12(2):e0171860. doi: 10.1371/journal.pone.0171860 (PMC5302316; doi:10.1371/journal.pone.0171860)

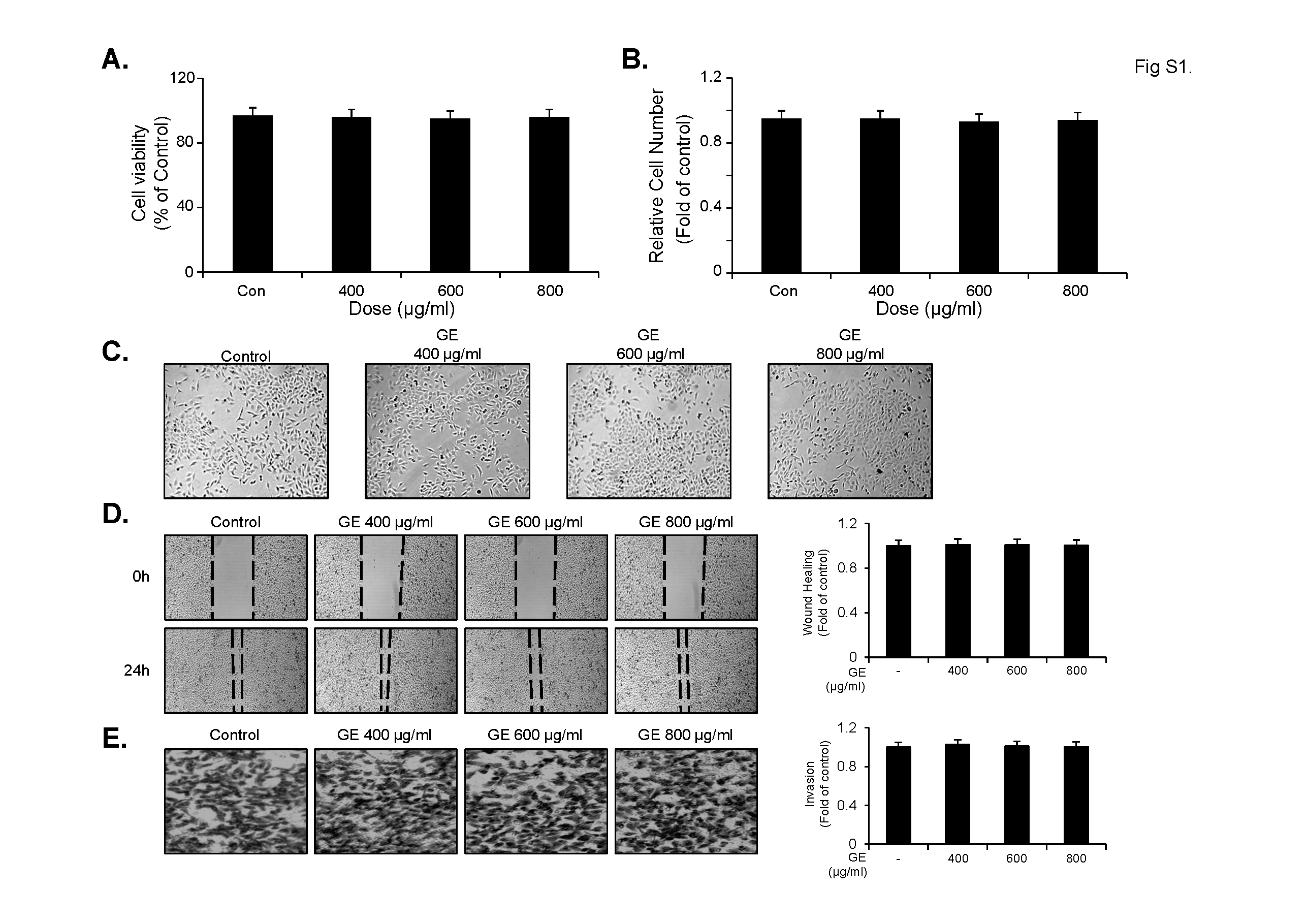

Supplement: S1 Fig — HUC cells were cultured with or without GE for 24 h. The cell viability and cell proliferation was estimated by both MTT (A) and viable cell counting assay (B). Results are expressed as mean ± SE from three different triplicate experiments. (C) The cell morphology generated from different concentrations of GE. Cellular images were captured with a phase contrast microscopy. (D, E) Wound-healing migration and invasion assay following treatment with GE in HUC cells. Results in bar graphs are expressed as mean ± SE from three different triplicate experiments. (TIFF) [file pone.0171860.s002.tiff]

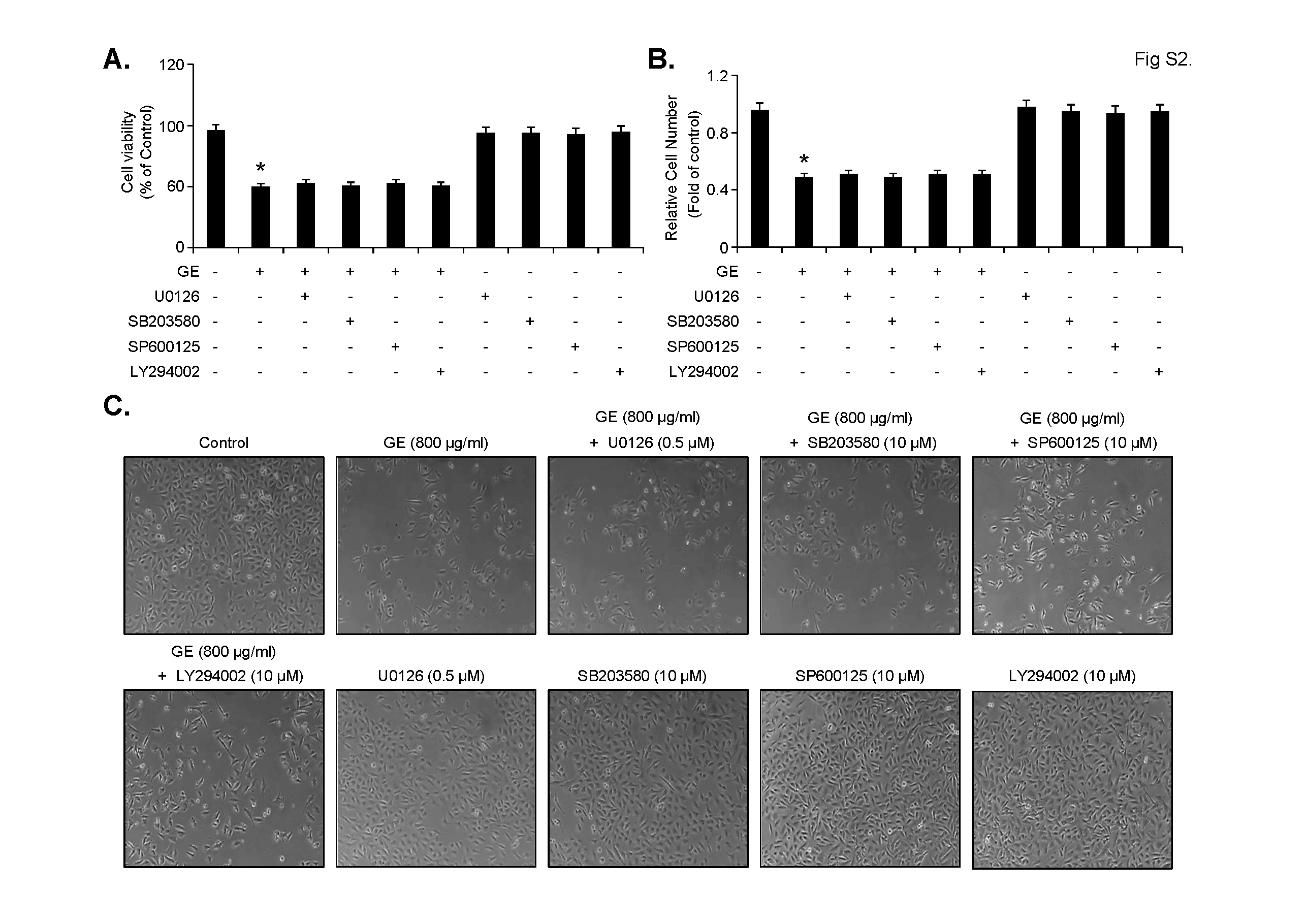

Supplement: S2 Fig — EJ cells were pre-treated with U0126 (0.5 μM), SB203580 (10 μM), SP600125 (10 μM), and LY 294002 (10 μM) for 40 min following treatment with GE (800 μg/ml). MTT (A) and viable cell counting assay (B) were performed to determine the cell viability and cell proliferation. (C) The cell morphology was photographed using a phase contrast microscopy. Results in bar graphs are reported as mean ± SE from three different triplicate experiments. *P<0.05 compared with the control. (TIFF) [file pone.0171860.s003.tiff]
